# Airway Emergencies Due to Anterior Mediastinal T-Lymphoblastic Lymphoma Managed With Planned Extracorporeal Membrane Oxygenation and Endotracheal Stent: A Case Report and Literature Review

**DOI:** 10.7759/cureus.21799

**Published:** 2022-02-01

**Authors:** Momoko Oyake, Souichi Suenobu, Michiyo Miyawaki, Yoshifumi Ohchi, Kenji Ihara

**Affiliations:** 1 Department of Pediatrics, Oita University Faculty of Medicine, Yufu, JPN; 2 Division of General Pediatrics and Emergency Medicine, Department of Pediatrics, Oita University Faculty of Medicine, Yufu, JPN; 3 Department of Thoracic and Breast Surgery, Oita University Faculty of Medicine, Yufu, JPN; 4 Department of Anesthesiology and Intensive Care, Oita University Faculty of Medicine, Yufu, JPN

**Keywords:** oncologic emergency, airway emergency, endotracheal stent, ecmo, mediastinal t-lbl

## Abstract

Anterior mediastinal tumors can occasionally cause acute respiratory failure by compressing the trachea and bronchi. In such cases, sedative muscle relaxants during tracheal intubation can cause fatal complete tracheal obstruction. We encountered a 15-year-old male patient with T-lymphoblastic lymphoma (T-LBL) of the anterior mediastinum. For his airway emergency due to the stenosis extended from the lower part of the trachea to the tracheal bifurcation, venovenous (VV) extracorporeal membrane oxygenation (ECMO) was introduced from the femoral vein under local anesthesia. After a short period of tracheal intubation management, an endotracheal stent (ES) was immediately placed in the lower trachea. We performed a needle biopsy, and he was diagnosed with T-LBL. Following the diagnosis, chemotherapy was introduced. The ES was able to secure sufficient tracheal diameter, and ECMO and ventilation were promptly discontinued. In the case of tracheal stenosis from the lower part of the trachea due to anterior mediastinal tumor, depending on the degree of stenosis, VV ECMO can be considered. Moreover, ES can lead to early weaning from VV ECMO and a ventilator.

## Introduction

Anterior mediastinal tumors can cause respiratory impairment due to airway obstruction, and sedative muscle relaxants can lead to potentially fatal complete tracheal obstruction. Therefore, extracorporeal membrane oxygenation (ECMO) can be considered in cases with lower trachea stenosis. However, complications can increase with prolonged ECMO and ventilation. The introduction of a temporary endotracheal stent (ES) together with ECMO can enable early weaning from ECMO and ventilator.

Here, we report the case of an adolescent male with an airway emergency caused by an anterior mediastinal tumor. A multidisciplinary team collaborated to introduce venovenous (VV) ECMO systematically and immediately inserted an ES to shorten the duration of intensive care.

## Case presentation

A 15-year-old male presented with dyspnea in the supine position. He had no previous history of tracheomalacia or tracheal stenosis. He was found to have a mediastinal tumor on chest X-ray and was immediately admitted to our hospital. The patient was naturally healthy and had no previous hospitalizations suggestive of tracheomalacia or tracheal stenosis.

**Figure 1:**
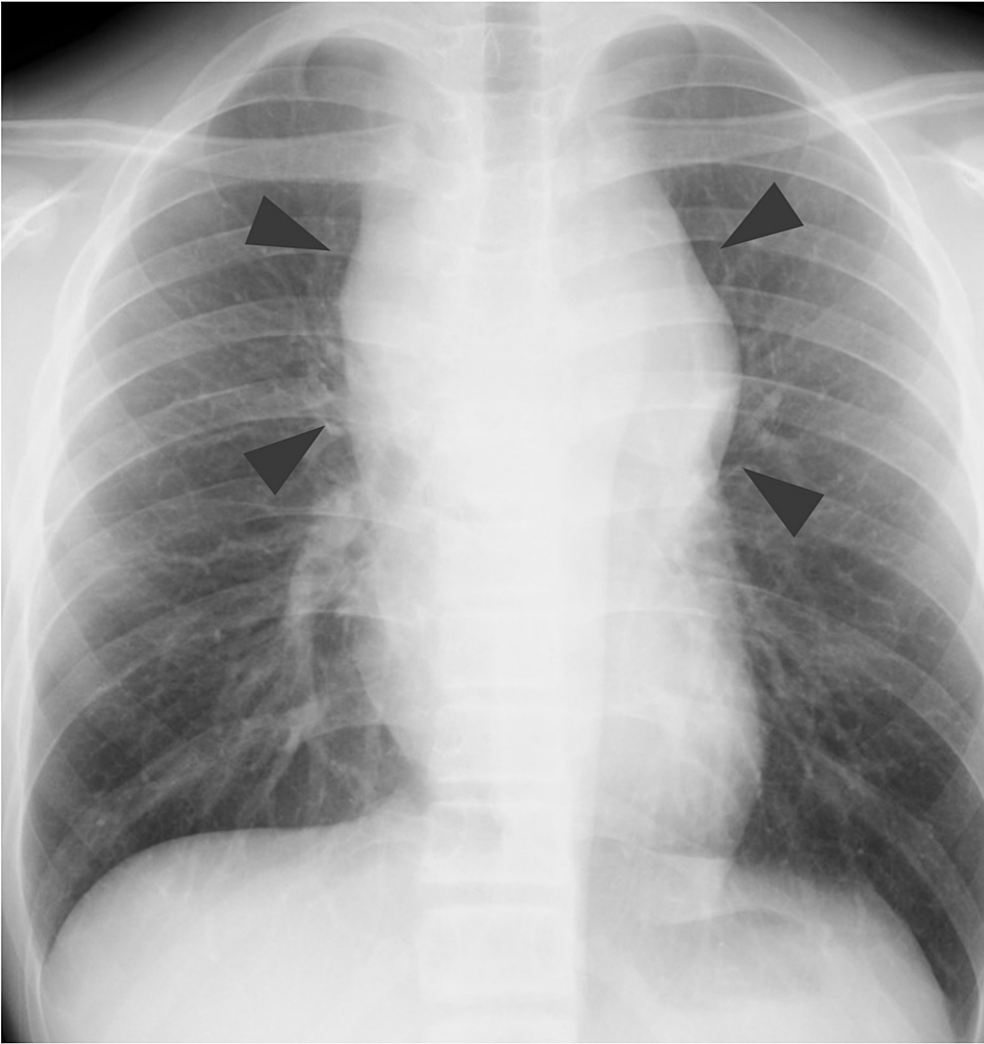
Chest X-ray (day 1). Enlargement of the upper mediastinum (arrowhead) and obscure tracheal shadow can be observed.

**Figure 2:**
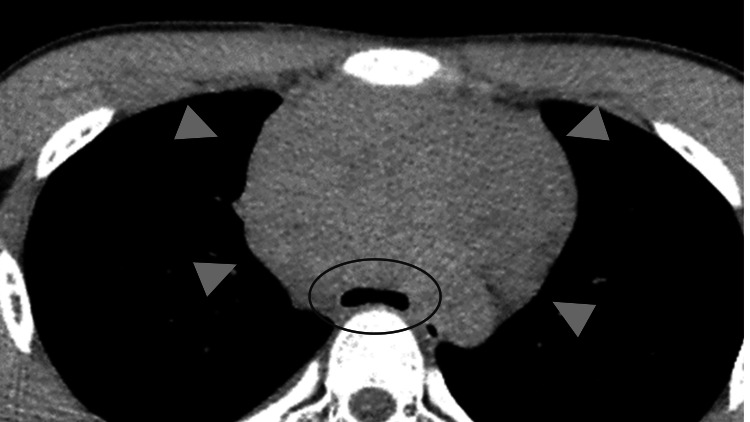
Contrast-enhanced CT chest (day 1). A 10 × 7 × 10 cm sized mass was found in the anterior mediastinum (arrowhead). The tracheal bifurcation was highly flattened (inside the circle). CT: computed tomography

On admission, he was 162.5 cm tall and weighed 45.1 kg. His SpO_2_ was 98% on room air, but he presented with orthopnea. Chest radiograph showed enlargement of the upper mediastinum (Figure 1), chest contrast-enhanced computed tomography (CT) of the chest demonstrated a 10 × 7 × 10 cm mass in the anterior mediastinum. Moreover, the trachea was highly flattened and compressed by the mass (Figure 2). Blood tests showed elevated lactate dehydrogenase (763 U/L) and soluble interleukin-2 receptor (1,024 U/mL), while complete blood counts were normal. The arterial blood gas analysis showed pH of 7.41, PO_2_ of 116 mmHg, PCO_2_ of 38 mmHg, and HCO_3_- of 23.7 mEq/L.

**Figure 3:**
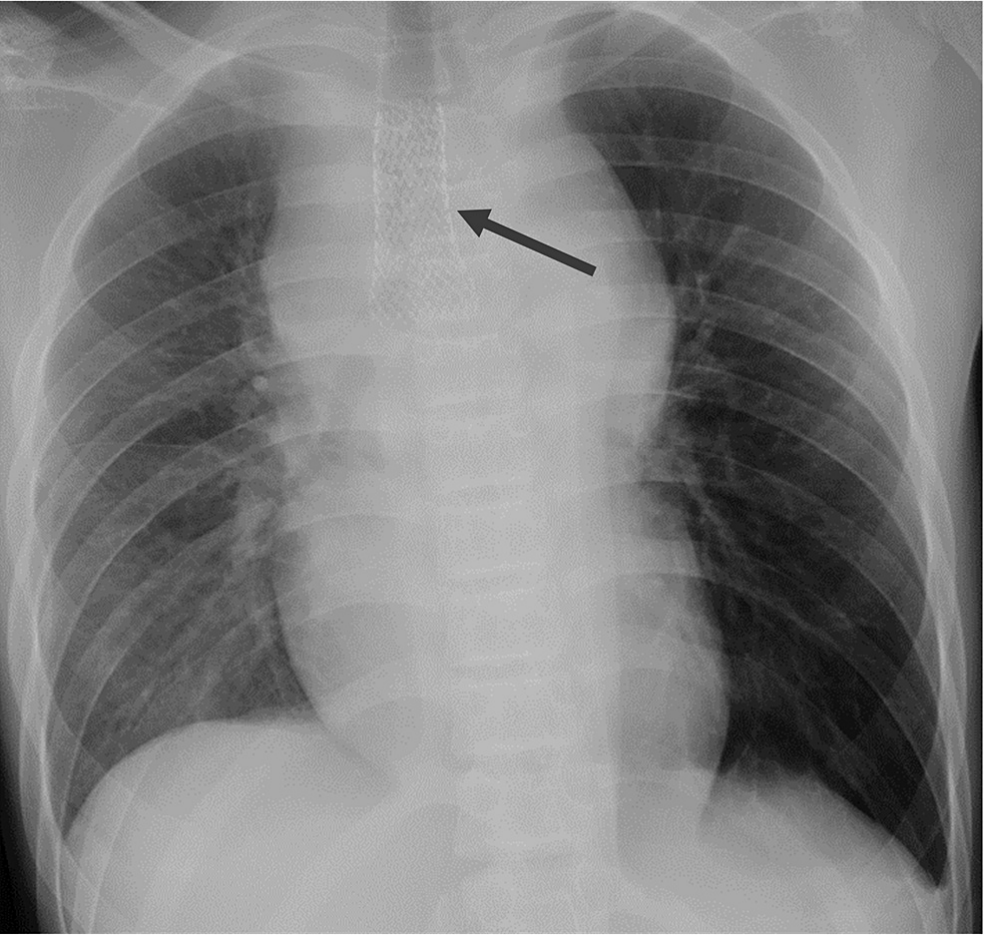
Chest X-ray (day 4). An endotracheal stent was placed in trachea (arrow).

**Figure 4:**
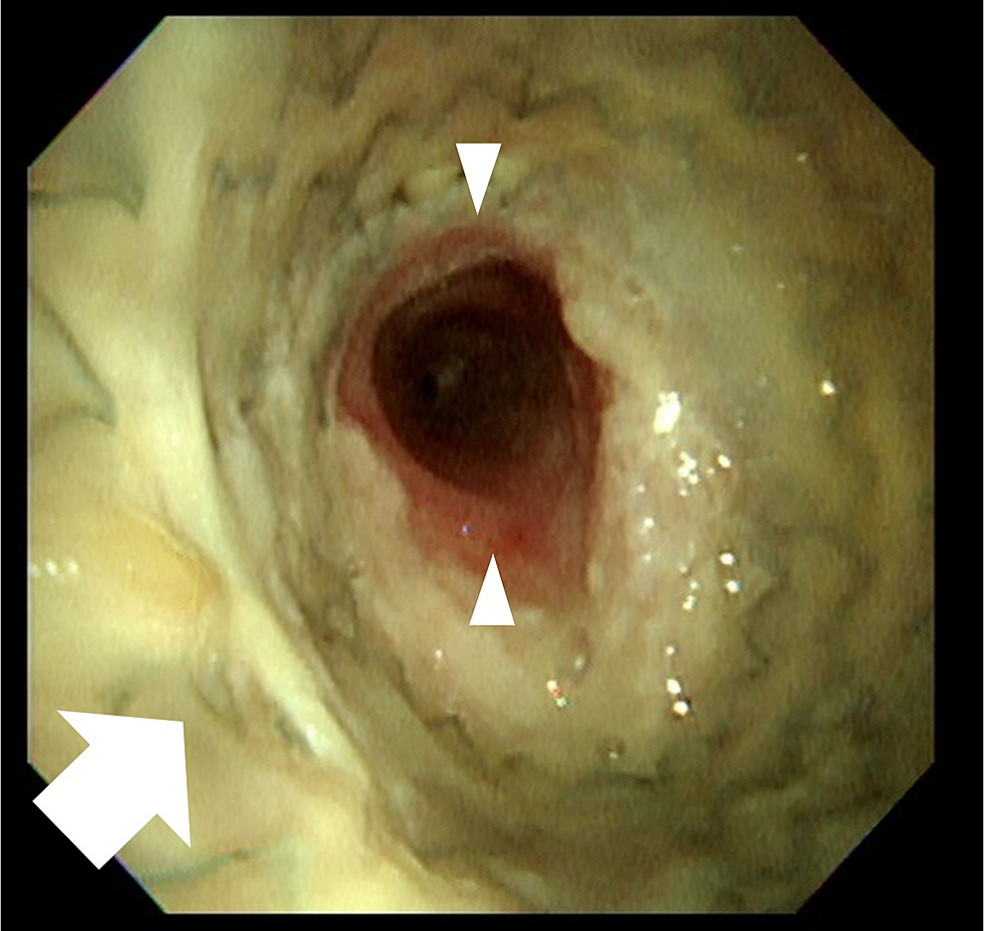
The bronchoscopic image of the trachea before ES removal. An ES (arrow) was properly placed, and the trachea was sufficiently patent (arrowhead). Although sputum adhesion was observed on the surface of the stent, there was no narrowing of the lumen due to it. ES: endotracheal stent

**Figure 5:**
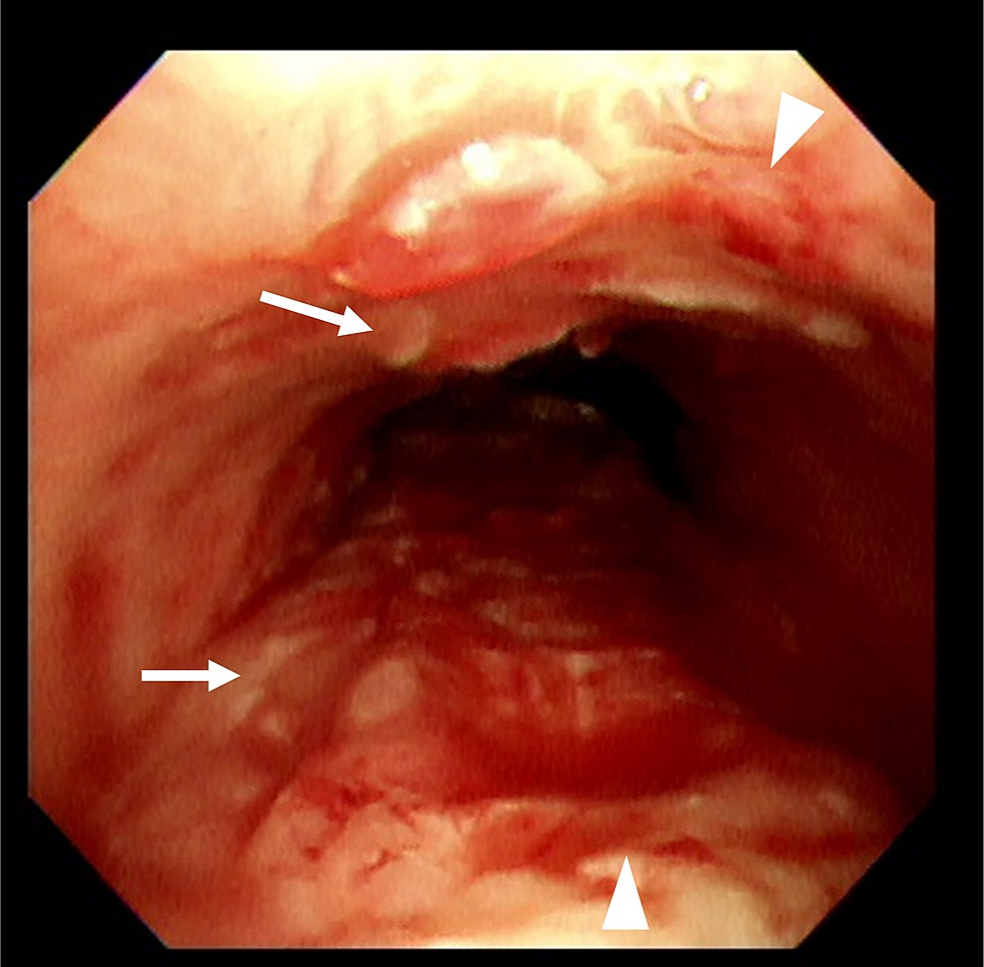
The bronchoscopic image of the trachea after ES removal. Immediately after the removal of ES, a small amount of bleeding (arrow) and localized detachment of the tracheal epithelium (arrowhead) were observed. ES: endotracheal stent

**Figure 6:**
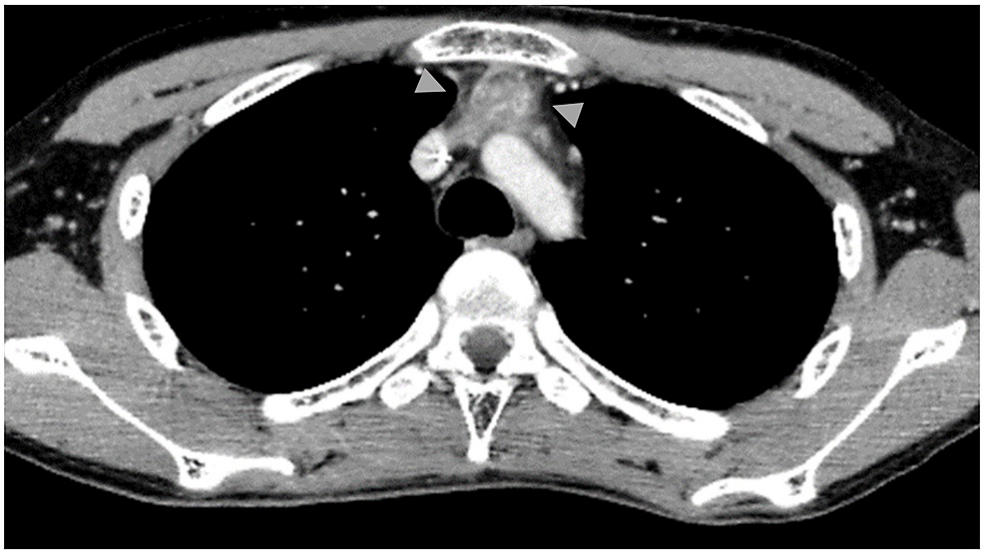
Contrast CT of the chest (day 96). The tumor diameter shrunk to 4.1 × 2.6 × 3.8 cm (arrowhead). CT: computed tomography

Due to an airway emergency, the patient was transferred to the intensive care unit (ICU). Because the stenosis extended from the lower part of the trachea to the tracheal bifurcation, ECMO and an ES were prepared as normal tracheal intubation was expected to be difficult. VV ECMO was introduced in the waking state using local anesthesia with dexmedetomidine and pentazocine. Subsequently, 22 and 18-Fr cannula were inserted into the left and right femoral vein for drawing and return, respectively. A BIO CUBE 6000® (NIPRO, Osaka, Japan) was used for the oxygenator, and an HLS cannula® (Maquet Getinge, Rastatt, Germany) was used for both devascularization and as a blood sending tube. The initial ECMO blood flow was 3 L/minute, with a sweep gas flow of 3.0 L/minute. Due to the planned short-term use of ECMO and the risk of bleeding from the tumor, anticoagulants were not administered. Once ECMO was introduced, we sedated the patient with propofol and rocuronium and performed tracheal intubation. There was no progressive respiratory impairment at this time, and tracheal intubation was performed with no obvious difficulty. A CT-guided biopsy and bone marrow examination were then performed, with no worsening of his condition noted. Finally, an ES (AERO® tracheobronchial stent; stent size, 18 × 60 mm; catalog number, 90123-202; Merit Medical Systems, UT, USA) was placed using a bronchoscope with support by fluoroscopy. While administering muscle relaxants, the airway became completely obstructed, and the SpO_2_ decreased to 70%; hence, we needed to increase the blood flow of the ECMO. After the ES had been inserted, the patient’s respiratory condition immediately stabilized (Figure 3). The time required was one hour for ECMO introduction and thirty minutes each for the CT-guided biopsy and ES insertion. The biopsy revealed a diagnosis of T-lymphoblastic lymphoma (T-LBL). He was sedated in the ICU with midazolam and dexmedetomidine, but no reduced oxygenation was observed. ECMO was weaned off in 23 hours and he was extubated. He left the ICU on day three, and the ES was removed on day 33 (Figures 4, 5). The thrombus in the internal jugular vein, detected on day six, disappeared after 56 days of anticoagulation. The chest CT on day 96 showed remarkable tumor shrinkage (Figure 6) and existence of local tracheal stenosis because of granulation. Chemotherapy for T-LBL was done according to the non-Hodgkin lymphoma-Berlin-Frankfurt-Münster-95 protocol. He has completed the chemotherapy protocol and has remained relapse-free for two years. Follow-up chest X-ray every four months has shown improvement in tracheal stenosis from 6 to 9 mm.

## Discussion

In cases of airway emergencies due to huge anterior mediastinal tumors, especially when the stenosis extends to the bifurcation of the airway, obtaining relief with conventional tracheal intubation can be difficult. In addition, sedation or general anesthesia to obtain biopsy specimens can cause complete airway obstruction. There is reportedly a significant risk of airway obstruction during general anesthesia if the bronchial diameter is competing for more than one-third of the lumen on CT [1]. In such cases, the use of ECMO as a respiratory aid is considered. An ECMO can provide stable circulation; however, complications such as bleeding, embolism, and infection with prolonged ECMO use have been reported [2,3]. In cases of hematological malignancies, early initiation of chemotherapy after a definitive diagnosis is critical to improving survival. An ECMO might be useful as a bridge to chemotherapy in patients with airway emergencies to increase the survival rate for such patients [4]; however, remission-induction chemotherapy causes neutropenia and an immunocompromised state, placing patients at risk of severe infections, such as ventilator-associated pneumonia. In such situations, ES may shorten the intensive care period [5]. Hence, planned ECMO and ES can be appropriate for respiratory management of these cases.

Table 1 summarizes the cases of ECMO in patients with hematological malignancies of the anterior mediastinal [5-15], including the present case, focusing on the intensive care period, airway management, and complications. The stenosis site extended from the lower part of the trachea to the bifurcation or the main bronchus in all cases, suggesting that ECMO introduction might be needed when respiratory management is difficult. Airway management with ECMO included conventional intubation in eight patients and ES in three patients, including our own. The other two patients were managed with ECMO alone. From the viewpoint of safe respiratory management, planned ECMO was performed in four cases, including two cases with ES and two managed with ECMO alone. In the other cases, ECMO introduction was unplanned: tracheal intubation became difficult in five cases, tracheal intubation itself was difficult in two cases, and complete airway obstruction during intubation, decreased oxygenation, and cardiopulmonary arrest during a biopsy occurred in one case each. In patients with planned ECMO, the intensive period, i.e., ventilation and ECMO periods, was shorter than that for unplanned ECMO. The longer the duration of ECMO, the more serious complications tended to be [3]. In addition, the ventilation duration was shorter in ES cases, suggesting that ES may be useful for preventing certain complications, such as ventilator-associated pneumonia. In two patients who did not require airway management, the duration of ECMO tended to be short, conferring a minimal risk of airway-associated infections; hence, ECMO alone might be suitable for patients without severe airway stenosis.

**Table 1:** A literature review of cases of ECMO in patients with hematological malignancies of the anterior mediastinal. ALL: acute lymphoblastic leukemia; Bil: bilateral; BL: Burkitt Lymphoma; CPAP: continuous positive airway pressure; CR: complete response; ECMO: extracorporeal membrane oxygenation; ES: endotracheal stent; F: female; Htx: hemothorax; LBL: Lymphoblastic lymphoma; Lt: left; MBCL: mature B-cell lymphoma; M: male; MB: main bronchus; ND: not described; NHL: non-Hodgkin lymphoma; PMBCL: primary mediastinal B-cell lymphoma; Pn: pneumonia; PR: partial response; TBF: tracheal bifurcation; TI: tracheal intubation; SVC: superior vena cava; VA: venoarterial; VT: venous thrombosis; VV: venovenous

Author	Leow et al. [5]	Lueck et al. [6]	Oto et al. [7]	Chao et al. [8]	Huang et al. [9]	Kim et al. [10]	Rotz et al. [11]	Aboud et al. [12]	Stewart et al. [13]	Wickiser et al. [14]	Wickiser [14]	Nokes et al. [15]	Our case
Year of publication	2021	2016	2014	2006	2010	2017	2020	2008	1998	2007	2007	2018	2022
Age (years)/Sex	29/M	20/M	40/M	21/F	15/M	20/M	18/M	43/F	19/M	11/M	4/M	49/F	15/M
Disease type	DLBCL	T-LBL	T-LBL	BL	T-ALL	DLBCL	PMBCL	NHL	LBL	T-ALL	T-NHL	MBCL	T-LBL
Narrowest part	TBF	Bil-MB	Lt-MB	Bil-MB	Lt-MB	ND	TBF	Lt-MB	TBF	TBF	ND	Bil-MB	TBF
Planned ECMO	No	No	No	No	No	No	No	No	No	Yes	Yes	Yes	Yes
ECMO type	VA	VA	VA	VV	VA	VA	VV	VV	VA	VA	VA	VV	VV
ECMO/Ventilator period	12 days	7 days	6 days	3 days	40 hours	2 days	40 days	5 days	2 days	5 days	3 days	1 day	23 hours
	ND	40 days	28 days	5 days	9 days	59 days	>40 days	13 days	10 days	None	None	None	1 day
Airway management	TI	TI	TI	TI (ES unsuccess)	TI →ES	TI	TI (ES migration)	TI (ES unsuccess)	TI	CPAP (Heliox)	(Heliox)	CPAP →ES	TI →ES
Complications of ECMO	ND	Htx, Pn	Skin abscess	None	None	ND	SVC syndrome	None	Lt-femoral ischemia	None	Lower limb VT	None	VT

In cases of hematologic malignancies, tumors may shrink rapidly with induction treatment; however, malignant lymphoma without tumor reduction for more than one month has been reported [9]. Therefore, if the degree of stenosis is severe, ES may be considered in addition to planned ECMO. Furthermore, in cases with successful ES, two of three patients were planned to receive ECMO, whereas all failed ES cases were treated with unplanned ECMO. Cases with stenosis in the lower part of the trachea can be safely managed by the planned introduction of ECMO [1], and ES may not fail. However, there are limitations of ES, such as the granulation of the trachea. Only a few reports have described ES for airway emergencies caused by hematological malignancies, and the timing of ES removal needs to be studied further with more cases. In addition, ES is contraindicated if a nonviable lung is present beyond the obstruction, or when vascular exclusion occurs [16,17]. We also need to be aware of the history of tracheal stenosis or tracheomalacia which makes the introduction of ES difficult. In these cases, it is necessary to increase the flow rate of ECMO to maintain oxygenation.

Furthermore, in this case, anticoagulant therapy was not performed when ECMO was used, resulting in the formation of a thrombus in the internal jugular vein; however, the thrombus disappeared by the following therapy. Hence, we conclude that the advantage of not using anticoagulants surpasses the risk of fatal airway bleeding during ECMO.

## Conclusions

Reviewing our experience and previous reports, we found that in airway emergencies caused by anterior mediastinal masses, it may be difficult to secure an airway by tracheal intubation alone, depending on the site and degree of airway stenosis. The planned introduction of ECMO in patients with a mediastinal mass caused by hematological malignancies can be considered, depending on the site of airway stenosis and stenotic degree (>50%), to allow respiratory management to be performed safely. In addition, ES can be considered as an option to prevent ECMO complications.
